# Effect of Porcine Akirin2 on Skeletal Myosin Heavy Chain Isoform Expression

**DOI:** 10.3390/ijms16023996

**Published:** 2015-02-12

**Authors:** Xiaoling Chen, Yanliu Luo, Bo Zhou, Zhiqing Huang, Gang Jia, Guangmang Liu, Hua Zhao, Zhouping Yang, Ruinan Zhang

**Affiliations:** 1 Key Laboratory for Animal Disease-Resistance Nutrition of China Ministry of Education, Institute of Animal Nutrition, Sichuan Agricultural University, Chengdu 611130, Sichuan, China; E-Mails: xlchen@sicau.edu.cn (X.C.); luoyanliu2014@163.com (Y.L.); zhoubo20091044@163.com (B.Z.); jiagang700510@163.com (G.J.); okliugm@gmail.com (G.L.); zhua666@126.com (H.Z.); ruinanzhang87@gmail.com (R.Z.); 2 College of Science, Sichuan Agricultural University, Chengdu 611130, Sichuan, China; E-Mail: yangzhouping007@163.com

**Keywords:** porcine Akirin2, myosin heavy chain, myoblast differentiation, myocyte enhancer factor 2, nuclear factor of activated T cells

## Abstract

Akirin2 plays an important role in skeletal myogenesis. In this study, we found that porcine *Akirin2* (*pAkirin2*) mRNA level was significantly higher in fast extensor digitorum longus (EDL) and longissimus lumborum (LL) muscles than in slow soleus (SOL) muscle of pigs. Overexpression of pAkirin2 increased the number of myosin heavy chain (MHC)-positive cells, indicating that pAkirin2 promoted myoblast differentiation. We also found that overexpression of pAkirin2 increased the mRNA expressions of *MHCI* and *MHCIIa* and decreased the mRNA expression of *MHCIIb*. Myocyte enhancer factor 2 (MEF2) and nuclear factor of activated T cells (NFAT) are the major downstream effectors of calcineurin. Here we also observed that the mRNA expressions of *MEF2C* and *NFATc1* were notably elevated by pAkirin2 overexpression. Together, our data indicate that the role of pAkirin2 in modulating MHCI and MHCIIa expressions may be achieved through calcineurin/NFATc1 signaling pathway.

## 1. Introduction

Skeletal muscle is comprised of muscle fibers, whose characteristics affect both lean meat production and meat quality [1,2]. Three main fiber types (slow oxidative type (I), fast oxiditave-glycotic type (IIa), and fast glycotic type (IIb)) can be distinguished according to their myosin ATPase stability after acid or alkali pretreatment [3]. Myosin ATPase is localized to the globular head of the myosin heavy chain (MHC) [4]. Therefore, MHC seems to represent the most appropriate marker for muscle fiber type. Four MHC isoforms of mammalian skeletal muscles are codified by four genes such as slow-twitch oxidative type I (MHCI), and three fast types, namely oxidative type IIa (MHCIIa), oxido-glycolytic type IIx (MHCIIx), and glycolytic type IIb (MHCIIb) [5,6]. Muscle fiber type composition, one of the main factors influencing meat quality, directly affects the muscle color, tenderness, and the content of intramuscular fat (IMF) in farm animals [7].

Improvement in meat quality is an important animal breeding goal, and consumers pay particular attention to meat quality. Regulation of muscle fiber type composition may be advantageous to achieve good meat quality in farm animals. Therefore, it is necessary to identify candidate genes that might contribute to improve meat quality by regulating muscle fiber type composition.

The *Akirin2* gene was previously reported to be associated with nuclear factor-κB (NF-κB) and to be involved in immune reactions, embryonic development and skeletal myogenesis [8,9,10,11,12]. The *Akirin2* gene has been previously shown to possess expression differences in musculus longissimus muscle between low-marbled and high-marbled steer groups and to be located within genomic region of a quantitative trait locus for marbling (the amount of IMF) [13]. Analysis of single nucleotide polymorphism (SNP) of *Akirin2* suggests that it is associated with marbling and may be useful for effective marker-assisted selection to increase the levels of marbling in Japanese black beef cattle [14,15]. A recent study also demonstrated that the SNP of *Akirin2* was significantly associated with longissimus muscle area and marbling score in Korean native cattle [16]. These studies suggested that the *Akirin2* gene may be associated with the content of IMF and affect the meat quality.

Until now, very little research has been conducted on the function of porcine Akirin2 (pAkirin2). In our previous study, we cloned the *pAkirin2* cDNA and examined its tissue distribution [17]. The *pAkirin2* cDNA was sub-cloned into prokaryotic expression vector pET28a(+), and target protein was successfully induced to express and was purified as expected [18]. Moreover, the purified recombinant pAkirin2 significantly increased the proliferation of C2C12 cells [18]. In the present study, we examined the *pAkirin2* mRNA expression in different types of muscle tissues of pigs and the effect of pAkirin2 on differentiation of C2C12 myoblasts. We also examined the effects of pAkirin2 on expressions of MHC isoform and oxidative muscle fiber genes in C2C12 myotubes.

## 2. Results

### 2.1. pAkirin2 mRNA Expression in Different Types of Muscle Tissues of Pigs

**Figure 1:**
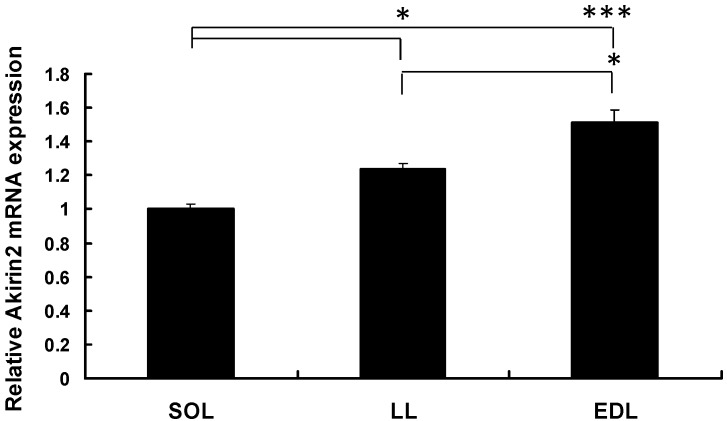
Relative *Akirin2* mRNA expression in different types of muscle tissues of pigs. Total RNA from slow soleus (SOL), longissimus lumborum (LL) and extensor digitorum longus (EDL) muscles of three healthy Duroc × Landrace × Yorkshire (DLY) pigs was used to perform the real-time quantitative PCR. Samples were performed in duplicate. The amount of *Akirin2* mRNA was normalized to the amount of *pβ-actin* mRNA. Data were presented as means ± SE (*n* = 3), in arbitrary units. * *p* < 0.05, *** *p* < 0.001.

The expression of *pAkirin2* mRNA in the soleus (SOL) muscle, longissimus lumborum (LL) muscle, and extensor digitorum longus (EDL) muscle of Duroc × Landrace × Yorkshire (DLY) pigs was assessed by real-time quantitative PCR. As shown in Figure 1, the expression of *pAkirin2* mRNA was higher in the EDL and LL muscles than in the SOL muscle of pigs.

**Figure 2:**
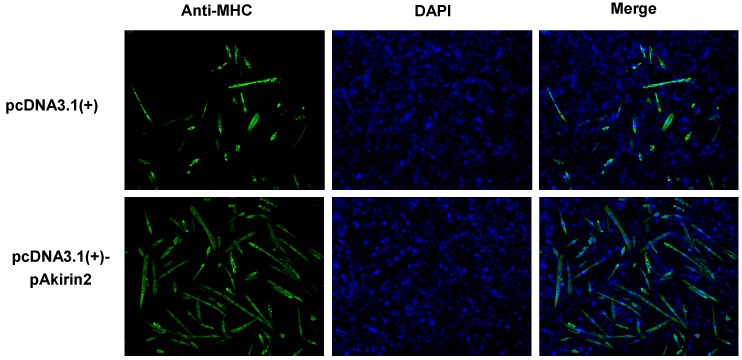
Effect of pAkirin2 on myoblast differentiation. C2C12 myoblasts were seeded in a 24-well plate at 1 × 10^4^ cells/well. The cells were transfected with 0.5 µg/well of the plasmid pcDNA3.1(+)-pAkirin2 or the empty vector pcDNA3.1(+) when they reached ~90% confluence and induced to differentiate for 5 days before analysis. Myosin heavy chain (MHC) expression was analyzed by immunofluorescence microscopy (DAPI staining also shown). The images are representative of the results obtained from two independent experiments. Magnification: ×100.

### 2.2. Effect of pAkirin2 on Myoblast Differentiation

MHC is a marker for later stages of myogenesis. To assess the function of pAkirin2 in myoblast differentiation, we introduced pAkirin2 into C2C12 myoblasts. As shown in Figure 2, overexpression of pAkirin2 increased the number of MHC-positive cells, suggesting that pAkirin2 promoted myoblast differentiation.

### 2.3. Effect of pAkirin2 on MHC Isoform Expression in C2C12 Myotubes

**Figure 3:**
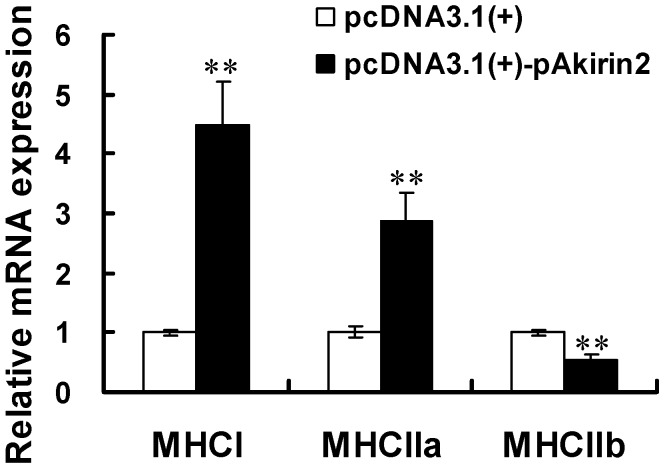
Effect of pAkirin2 on MHC isoform expression in C2C12 myotubes. C2C12 myoblasts were cultured and transfected as in Figure 2. Two days after the transfection, the mRNA levels of *MHCI*, *MHCIIa* and *MHCIIb* were determined using real-time quantitative PCR. Samples were performed in duplicate. The amount of *MHCI*, *MHCIIa* and *MHCIIb* mRNA were normalized to the amount of *GAPDH* mRNA and *mβ-actin* mRNA. Data were presented as means ± SE (*n* = 3). ** *p* < 0.01.

We evaluated the effect of pAkirin2 on MHC isoform expression. As shown in Figure 3, the mRNA expressions of *MHCI* and *MHCIIa* were significantly increased, whereas the mRNA expression of *MHCIIb* was significantly decreased, in C2C12 myotubes transfected with the plasmid pcDNA3.1(+)-pAkirin2.

### 2.4. Effect of pAkirin2 on Oxidative Muscle Fiber Gene Expression in C2C12 Myotubes

**Figure 4:**
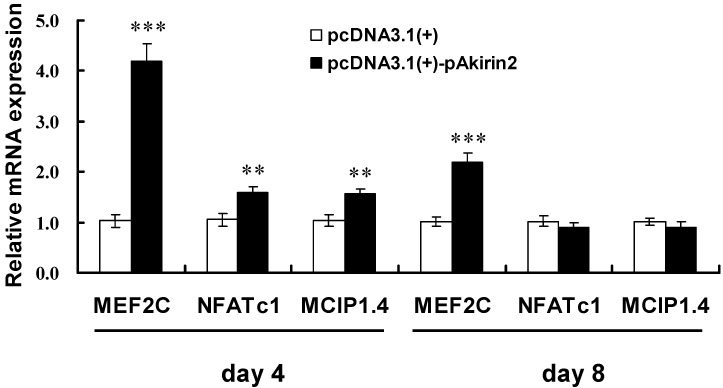
Effect of pAkirin2 on oxidative muscle fiber gene expression in C2C12 myotubes. C2C12 myoblasts were cultured and transfected as in Figure 2. After a transfection of 4 and 8 days, the mRNA levels of *MEF2C*, *NFATc1* and *MCIP1.4* were determined by real-time quantitative PCR. Samples were performed in duplicate. The amount of *MEF2C*, *NFATc1* and *MCIP1.4* mRNA were normalized to the amount of *GAPDH* mRNA and *mβ-actin* mRNA. Data were presented as means ± SE (*n* = 3). ** *p* < 0.01, *** *p* < 0.001.

To explore the effect of pAkirin2 on oxidative muscle fiber gene expression, we measured the expressions of MEF2C (myocyte enhancer factor-2C), NFATc1 (nuclear factor of activated T cells, cytoplasmic 1), and MCIP1.4 (modulatory calcineurin interacting protein 1 exon 4 isform) by real-time quantitative PCR. The data obtained showed that overexpression of pAkirin2 significantly increased the mRNA expressions of *MEF2C*, *NFATc1* and *MCIP1.4* in C2C12 myotubes on day 4 (Figure 4). On day 8, overexpression of pAkirin2 strongly increased *MEF2C* mRNA expression but had no significant effect on *NFATc1* and *MCIP1*.4 mRNA expressions (Figure 4).

## 3. Discussion

Skeletal muscle is composed of three groups of muscle fibers (slow, fast and intermediate). In animal production industry, specific combinations of fast and slow muscles affect the meat quality [19]. In the present study, we determined the expression of *pAkirin2* mRNA in the fast EDL and LL muscles and slow SOL muscle of pigs. We found that *pAkirin2* mRNA expression was most abundant in the EDL muscle, followed by the LL muscle, and to a lesser extent in the SOL muscle. Our data suggested that the expression of *pAkirin2* was significantly higher in fast muscles than in slow muscles and might be closely related to the expression of muscle fiber type-related genes.

Oxidative fibers (type I and type IIa) seem to be positively related to the color, water-holding capacity and tenderness of meat [20]. Type IIb fibers have a larger diameter than other fiber types and contribute to increase in muscle mass [21]. However, in pigs, higher percentage of type IIb fiber has been shown to be negatively related to pH_45 min_ and positively to drip loss and *R*-value (adenine/inosine ratio), thereby resulting in reducing the meat quality [20,22]. In this study, we found that *MHCI* and *MHCIIa* were upregulated, whereas *MHCIIb* was downregulated, by pAkirin2 overexpression. Although the result is in contradiction to the finding that *pAkirin2* mRNA is higher expressed in fast than in slow muscle of pigs, the reason for this remains unclear. Taken together, these results suggested that the *pAkirin2* gene may have an important function in regulating meat quality by affecting fiber type-specific gene expression.

The calcineurin signaling pathway has been implicated in the regulation of slow skeletal muscle fiber gene expression [23,24]. MEF2 and NFAT proteins are the major downstream effectors of calcineurin [23,24,25,26,27]. NFAT is one of the primary cofactors for MEF2 [28]. Elevated calcium signaling is essential for optimal expression of the MHCI via calcineurin/NFAT pathway [24,29,30,31]. In the present study, overexpression of pAkirin2 increased the expression of transcription factors MEF2C and NFATc1, both involved in the regulation of oxidative muscle fiber genes. It should be noted that activated NFATc1 (dephosphorylated in the nuclear) is known to promote slow fiber type-specific gene expression, whereas inactivated NFATc1 (phosphorylated in the cytoplasma) can also be found in fast muscles [32]. In addition, NFATc1 can interact with MEF2 isoforms in slow fiber type-specific gene expression depending on the promoter context. For example, together with NFATc1, the isoform MEF2D regulates the slow MHCI promoter [33], but the isoform MEF2C is involved in fast MHCIIa and fast MHCIIx promoter activation [34,35]. Because activation of MEF2C at target promoters occurs primarily via phosphorylation, increased expression is not necessary. However, since activation of the calcineurin/NFATc1 signaling pathway is accompanied by the increase of certain NFATc1 mRNA and protein levels [36] and the primers used for *NFATc1* in this study include the induced isoforms, the observed increase in *NFATc1* mRNA expression by pAkirin2 overexpression can be utilized as an indication for activation of the calcineurin/NFATc1 signaling pathway in this experimental setup. MCIP1.4 is a direct downstream target of the calcineurin/NFAT pathway, which has recently been renamed as regulator of calcineurin 1 (RCAN1) [37]. MCIP1.4 was reported to increase the number of MHCI-expressing slow fibers [38]. Here we also observed that overexpression of pAkirin2 enhanced the expression of MCIP1.4. Together, our results suggest that the role of pAkirin2 in regulating MHCI and MHCIIa expressions may be achieved through calcineurin/NFATc1 signaling pathway.

## 4. Materials and Methods

### 4.1. Animals and Tissue Sample Collection

Three 10-week-old female DLY pigs (body weight of 31.27 ± 0.18 kg) were slaughtered in a humane manner according to protocols approved by the Animal Care Advisory Committee of Sichuan Agricultural University under permit No. YYS130125. The SOL, LL and EDL muscles were removed and immediately snap frozen in liquid nitrogen before being stored at −80 °C for RNA isolation.

### 4.2. RNA Isolation and Reverse Transcription

Total RNA was isolated using RNAiso Plus reagent (TaKaRa, Dalian, China) according to the manufacturer’s instructions. The concentrations of total RNA were quantified using a Beckman DU–800 spectrophotometer (Beckman Coulter, Fullerton, CA, USA). cDNA was synthesized from one microgram of total RNA using a PrimeScript^®^ RT reagent Kit with gDNA Eraser (TaKaRa) according to the manufacturer’s protocols. The first-strand cDNA was subsequently used as a template for real-time quantitative PCR.

### 4.3. Cell Culture and Transfection

Mouse C2C12 myoblasts (CRL-1772) were obtained from American Type Culture Collection (ATCC, Rockville, MD, USA). The cell line was grown in Dulbecco modified Eagle medium (DMEM) (Invitrogen, Carlsbad, CA, USA) supplemented with 10% fetal bovine serum (FBS) (Invitrogen) and 100 U/mL penicillin and 100 μg/L streptomycin (ATCC) at 37 °C in a 5% CO_2_ atmosphere. The cells were induced to differentiate with DMEM containing 2% horse serum (ATCC) when they reached approximately 90% confluence. Medium was then renewed every day before analysis. C2C12 cells were transfected with pcDNA3.1(+)-pAkirin2 [17] or pcDNA3.1(+) using Lipofectamine 2000 (Invitrogen) according to the manufacturer’s instruction.

### 4.4. Real-Time Quantitative PCR

Real-time quantitative PCR was performed on a 7900HT Real-time PCR system (384-cell standard block) (Applied Biosystems, Foster, CA, USA) in a final volume of 10 μL. The gene specific primers used are listed in Table 1. The PCR mixture consisted of 1 μL of the first-strand cDNA sample, 1 μL each of forward and reverse primers from 10 μM stocks, 2 μL DEPC-treated water, and 5 μL of SYBR select Master Mix (Applied Biosystems). The initial denaturation step at 95 °C for 10 min was followed by 45 cycles of denaturation for 15 s at 95 °C, and annealing and extension for 30 s at 60 °C. Each primer pair used yielded a single peak in the melting curve and a single band with the expected size in agarose gel. Identities of the PCR products were confirmed by DNA sequencing. Data analysis was performed using the comparative *C*t method [39] with *GAPDH* and/or *β-actin* as an endogenous control.

**Table 1:** List of genes, primer sequences, GenBank accession numbers, and product sizes in this study.

Gene Name	Primer	Sequence	GenBank Accession No.	Product Size (bp)
*MHCI*	Forward	5'-CTTCTACAGGCCTGGGCTTAC-3'	**NM_080728**	128
	Reverse	5'-CTCCTTCTCAGACTTCCGCAG-3'		
*MHCIIa*	Forward	5'-TTCCAGAAGCCTAAGGTGGTC-3'	**NM_001039545**	94
	Reverse	5'-GCCAGCCAGTGATGTTGTAAT-3'		
*MHCIIb*	Forward	5'-CTTGTCTGACTCAAGCCTGCC-3'	**NM_010855**	158
	Reverse	5'-TCGCTCCTTTTCAGACTTCCG-3'		
*Akirin2*	Forward	5'-GATGGGACTGGATTATCGC-3'	**JN227885**	154
	Reverse	5'-GCACAAGATGAGTATGCGG-3'		
*MCIP1.4*	Forward	5'-CCGTTGGCTGGAAACAAG-3'	**NM_019466**	153
	Reverse	5'-GGTCACTCTCACACACGTGG-3'		
*NFATc1*	Forward	5'-AATAACATGCGAGCCATCATC-3'	**AF239169**	109
	Reverse	5'-TCACCCTGGTGTTCTTCCTC-3'		
*MEF2C*	Forward	5'-GATCTCCGCGTTCTTATCCC-3'	**L13171**	91
	Reverse	5'-CCAATGACTGAGCCGACTG-3'		
*GAPDH*	Forward	5'-AGGGCATCTTGGGCTACAC-3'	**NM_008084**	211
	Reverse	5'-TGGTCCAGGGTTTCTTACTCC-3'		
*mβ-actin*	Forward	5'-CCTTCCTTCTTGGGTATGGA-3'	**NM_007393**	88
	Reverse	5'-GGTCTTTACGGATGTCAACG-3'		
*pβ-actin*	Forward	5'-CCACGAAACTACCTTCAACTCC-3'	**DQ845171**	132
	Reverse	5'-GTGATCTCCTTCTGCATCCTGT-3'		

### 4.5. Cell Immunofluorescence Assay

Cells were fixed in 4% formaldehyde in phosphate-buffered saline (PBS) for 20 min and permeabilised with 0.1% Triton X-100 in PBS for 20 min. Cells were then blocked in 5% bovine serum albumin (BSA) for 30 min and incubated with MHC antibody (Santa Cruz Biotechnology; sc-20641, 1:100, Santa Cruz, CA, USA) at 4 °C overnight. The cells were rinsed with PBS and incubated with FITC-conjugated secondary antibody (Santa Cruz Biotechnology; 1:200) for 1 h at room temperature. To stain the nuclei, the cells were incubated in the DNA stain 4'6-diamidino-2-phenylindole (DAPI) for 10 min. Images were captured using a Nikon Eclipse TS100 inverted fluorescence microscope (Nikon, Tokyo, Japan).

### 4.6. Statistical Analysis

All data were expressed as means ± SE (standard error) and were analyzed using SPSS11.5 software (SPSS Inc., Chicago, IL, USA). Group differences were analyzed by ANOVA and the differences between groups were considered to be statistically significant when *p* < 0.05.

## 5. Conclusions

In conclusion, we found that *pAkirin2* mRNA expression level is higher in the EDL and LL muscles than in the SOL muscle. Overexpression of pAkirin2 promoted differentiation of C2C12 myoblasts. Moreover, we also provided the evidence that overexpression of pAkirin2 led to up-regulation of MHC isoform (MHCI and MHCIIa) and oxidative muscle fiber gene (MEF2C, NFATc1 and MCIP1.4) expressions. This study contributes to understand the role of Akirin2 in the regulation of muscle fiber types using pig as a model organism and helps to explore the key genes regulating meat quality.

## References

[1] Brocks L., Klont R.E., Buist W., de Greef K., Tieman M., Engel B. (2000). The effects of selection of pigs on growth rate *vs*. leanness on histochemical characteristics of different muscles. J. Anim. Sci..

[2] Ryu Y.C., Kim B.C. (2006). Comparison of histochemical characteristics in various pork groups categorized by postmortem metabolic rate and pork quality. J. Anim. Sci..

[3] Brooke M.H., Kaiser K.K. (1970). Muscle fiber types: How many and what kind?. Arch. Neurol..

[4] Levitsky D.I. (2004). Actomyosin systems of biological motility. Biochemistry.

[5] Lefaucheur L., Ecolan P., Plantard L., Gueguen N. (2002). New insights into muscle fiber types in the pig. J. Histochem. Cytochem..

[6] Kim G.D., Kim B.W., Jeong J.Y., Hur S.J., Cho I.C., Lim H.T., Joo S.T. (2013). Relationship of carcassweight tomuscle fiber characteristics and pork quality of crossbred (Korean native black pig × Landrace) F2 pigs. Food Bioprocess Technol..

[7] Lee S.H., Joo S.T., Ryu Y.C. (2010). Skeletal muscle fiber type and myofibrillar proteins in relation to meat quality. Meat Sci..

[8] Goto A., Matsushita K., Gesellchen V., Chamy L.E., Kuttenkeuler D., Takeuchi O., Hoffmann J.A., Akira S., Boutros M., Reichhart J. (2008). Akirins are highly conserved nuclear proteins required for NF-κB-dependent gene expression in drosophila and mice. Nat. Immunol..

[9] Beutler B., Moresco E.M.Y. (2008). Akirins versus infection. Nat. Immunol..

[10] Macqueen D.J., Johnston I.A. (2009). Evolution of the multifaceted eukaryotic *Akirin* gene family. BMC Evol. Biol..

[11] Macqueen D.J., Bower N.I., Johnston I.A. (2010). Positioning the expanded *Akirin* gene family of Atlantic salmon within the transcriptional networks of myogenesis. Biochem. Biophys. Res. Commun..

[12] Chen X.L., Huang Z.Q., Wang H., Jia G., Liu G.M., Guo X.L., Tang R.Y., Long D.B. (2013). Role of Akirin in skeletal myogenesis. Int. J. Mol. Sci..

[13] Sasaki Y., Nagai K., Nagata Y., Doronbekov K., Nishimura S., Yoshioka S., Fujita T., Shiga K., Miyake T., Taniguchi Y. (2006). Exploration of genes showing intramuscular fat deposition-associated changes in musculus longissimus muscle. Anim. Genet..

[14] Sasaki S., Yamada T., Sukegawa S., Miyake T., Fujita T., Morita M., Ohta T., Takahagi Y., Murakami H., Morimatsu F. (2009). Association of a single nucleotide polymorphism in *Akirin2* gene with marbling in Japanese Black beef cattle. BMC Res. Notes.

[15] Watanabe N., Satoh Y., Fujita T., Ohta T., Kose H., Muramatsu Y., Yamamoto T., Yamada T. (2011). Distribution of allele frequencies at *TTN g.231054C>T*, *RPL27A g.3109537C>T* and *Akirin2 c.*188G>A* between Japanese black and four other cattle breeds with differing historical selection for marbling. BMC Res. Notes.

[16] Kim H., Lee S.K., Hong M.W., Park S.R., Lee Y.S., Kim J.W., Lee H.K., Jeong D.K., Song Y.H., Lee S.J. (2013). Association of a single nucleotide polymorphism in the *Akirin2* gene with economically important traits in Korean native cattle. Anim. Genet..

[17] Chen X.L., Huang Z.Q., Jia G., Wu X.Q., Wu C.M. (2012). Molecular cloning, tissue distribution, and functional analysis of porcine Akirin2. Anim. Biotechnol..

[18] Chen X., Huang Z., Zhou B., Wang H., Jia G., Qiao J. (2014). Expression and purification of porcine Akirin2 in *Escherichia coli*. Turk. J. Biol..

[19] Hwang Y.H., Kim G.D., Jeong J.Y., Hur S.J., Joo S.T. (2010). The relationship between muscle fiber characteristics and meat quality traits of highly marbled Hanwoo (Korean native cattle) steers. Meat Sci..

[20] Chang K.C., da Costa N., Blackley R., Southwood O., Evans G., Plastow G., Wood J.D., Richardson R.I. (2003). Relationships of myosin heavy chain fibre types to meat quality traits in traditional and modern pigs. Meat Sci..

[21] Ryu Y.C., Choi Y.M., Lee S.H., Shin H.G., Choe J.H., Kim J.M., Hong K.C., Kim B.C. (2008). Comparing the histochemical characteristics and meat quality traits of different pig breeds. Meat Sci..

[22] Ryu Y.C., Kim B.C. (2005). The relationship between muscle fiber characteristics, postmortem metabolic rate, and meat quality of pig *longissimus dorsi* muscle. Meat Sci..

[23] Chin E.R., Olson E.N., Richardson J.A., Yang Q., Humphries C., Shelton J.M., Wu H., Zhu W., Bassel-Duby R., Williams R.S. (1998). A calcineurin-dependent transcriptional pathway controls skeletal muscle fiber type. Genes Dev..

[24] Bassel-Duby R., Olson E.N. (2009). Signaling pathways in skeletal muscle remodeling. Annu. Rev. Biochem..

[25] Olson E.N., Williams R.S. (2000). Calcineurin signaling and muscle remodeling. Cell.

[26] Wu H., Rothermel B., Kanatous S., Rosenberg P., Naya F.J., Shelton J.M., Hutcheson K.A., DiMaio J.M., Olson E.N., Bassel-Duby R. (2001). Activation of MEF2 by muscle activity is mediated through a calcineurin dependent pathway. EMBO J..

[27] Mu X., Brown L.D., Liu Y., Schneider M.F. (2007). Roles of the calcineurin and CaMK signaling pathways in fast-to-slow fiber type transformation of cultured adult mouse skeletal muscle fibers. Physiol. Genomics.

[28] Blaeser F., Ho N., Prywes R., Chatila T.A. (2000). Ca^2+^-dependent gene expression mediated by MEF2 transcription factors. J. Biol. Chem..

[29] Abbott K.L., Friday B.B., Thaloor D., Murphy T.J., Pavlath G.K. (1998). Activation and cellular localization of the cyclosporine A-sensitive transcription factor NF-AT in skeletal muscle cells. Mol. Biol. Cell.

[30] Im S.H., Rao A. (2004). Activation and deactivation of gene expression by Ca^2+^/calcineurin-NFAT-mediated signaling. Mol. Cells.

[31] Meissner J.D., Freund R., Krone D., Umeda P.K., Chang K.C., Gros G., Scheibe R.J. (2011). Extracellular signal-regulated kinase 1/2-mediated phosphorylation of p300 enhances myosin heavy chain I/β gene expression via acetylation of nuclear factor of activated T cells c1. Nucleic Acids Res..

[32] Calabria E., Ciciliot S., Moretti I., Garcia M., Picard A., Dyar K.A., Pallafacchina G., Tothova J., Schiaffino S., Murgia M. (2009). FAT isoforms control activity-dependent muscle fiber type specification. Proc. Natl. Acad. Sci. USA.

[33] Meissner J.D., Umeda P.K., Chang K.C., Gros G., Scheibe R.J. (2007). Activation of the β myosin heavy chain promoter by MEF-2D, MyoD, p300, and the calcineurin/NFATc1 pathway. J. Cell. Physiol..

[34] Allen D.L., Leinwand L.A. (2002). Intracellular calcium and myosin isoform transitions. Calcineurin and calcium-calmodulin kinase pathways regulate preferential activation of the IIa myosin heavy chain promoter. J. Biol. Chem..

[35] Meissner J.D., Chang K.C., Kubis H.P., Nebreda A.R., Gros G., Scheibe R.J. (2007). The p38α/β mitogen-activated protein kinases mediate recruitment of CREB-binding protein to preserve fast myosin heavy chain IId/x gene activity in myotubes. J. Biol. Chem..

[36] Serfling E., Chuvpilo S., Liu J., Höfer T., Palmetshofer A. (2006). NFATc1 autoregulation: A crucial step for cell-fate determination. Trends Immunol..

[37] Davies K.J., Ermak G., Rothermel B.A., Pritchard M., Heitman J., Ahnn J., Henrique-Silva F., Crawford D., Canaider S., Strippoli P. (2007). Renaming the *DSCR1/Adapt78* gene family as RCAN: Regulators of calcineurin. FASEB J..

[38] Fenyvesi R., Rácz G., Wuytack F., Zádor E. (2004). The calcineurin activity and MCIP1.4 mRNA levels are increased by innervation in regenerating soleus muscle. Biochem. Biophys. Res. Commun..

[39] Livak K.J., Schmittgen T.D. (2001). Analysis of relative gene expression data using real-time quantitative PCR and the 2^–ΔΔ*C*t^ method. Methods.

